# Cause analysis of the liner dissociation of a customized reverse shoulder prosthesis based on finite element analysis

**DOI:** 10.3389/fbioe.2022.1038116

**Published:** 2022-12-02

**Authors:** Qian Wan, Aobo Zhang, Haotian Bai, Yang Liu, Hao Chen, Xin Zhao, Xiaonan Wang, Qing Han, Jincheng Wang

**Affiliations:** Department of Orthopedics, The Second Hospital of Jilin University, Changchun, China

**Keywords:** reverse total shoulder arthroplasty, finite element analysis, polyethylene liner dissociation, shoulder dislocation, fixation, impingement

## Abstract

**Background:** Dissociation of the polyethylene liner after reverse shoulder arthroplasty could cause shoulder dislocation that could not achieve closed reduction. The cause of liner dissociation is currently unclear.

**Method:** Non-homogeneous model of the bone was constructed and dynamic finite element analysis was utilized to simulate the impingement of the polyethylene liner and scapula during humeral adduction. The stress distribution of the fixation claws, their degree of deformation (DOD), and the stress of the impingement sites in three initial humeral postures (neutral, 30° flexion, and 30° extension) were measured and analyzed. The influence of the liner material stiffness was also investigated.

**Result:** The impingement stress on the liner and scapula was 100–200 MPa, and different humeral postures caused different locations of impingement points. The fixation claws’ maximum principal stress (MPS) results were below 5 MPa. In the connection area between some fixation claws and the liner, compressive stresses on the inside and tensile stresses on the outside were observed, which showed that the fixation claws were prone to deform toward the center direction. The maximum DOD results of three initial humeral postures (neutral, 30° flexion, and 30° extension) were 3.6%, 2.8%, and 3.5%, respectively. The maximum DOD results of neutral initial humeral posture were 0.51% and 11.4% when the elastic modulus of the liner was increased and decreased by a factor of 10, respectively.

**Conclusion:** The humeral adduction impingement could lead to the deformation of the claw-shaped liner fixation structure, which might be one of the reasons for the liner dissociation. The increased stiffness of the liner material helped to reduce the deformation of the fixation structure.

## 1 Introduction

The reverse shoulder prosthesis was invented to compensate for the loss of rotator cuff function in the 1970s, which was the opposite design of the anatomic shoulder prosthesis (Hansen and Routman, 2019). The incidence of primary reverse total shoulder arthroplasty (RTSA) has increased substantially in recent years. It reached 19.3 cases per 100,000 in 2017 in the United States, which was 2.64 times the incidence in 2012 (Best et al., 2021). However, the design of reverse shoulder prosthesis frequently causes impingement between the scapular pillar and the polyethylene liner of the humeral component (Friedman et al., 2019). This impingement was considered related to scapular notching, polyethylene wear, and glenoid implant loosening (Mollon et al., 2017; Griffiths et al., 2020). Efforts were made to reduce impingement by altering the design or placement of the prosthesis. Inferior tilt of the glenosphere, lateral offset of the glenosphere, placing the glenosphere inferiorly, and decreasing the neck shaft angle of the humeral component are considered to decrease the tendency of the impingement. However, these parameters are not independent, they influence each other and affect the deltoid moment, the stress on the prosthesis, etc (Ackland et al., 2015). Therefore, the optimal prosthesis design and placement are not yet conclusive.

**FIGURE 1:**
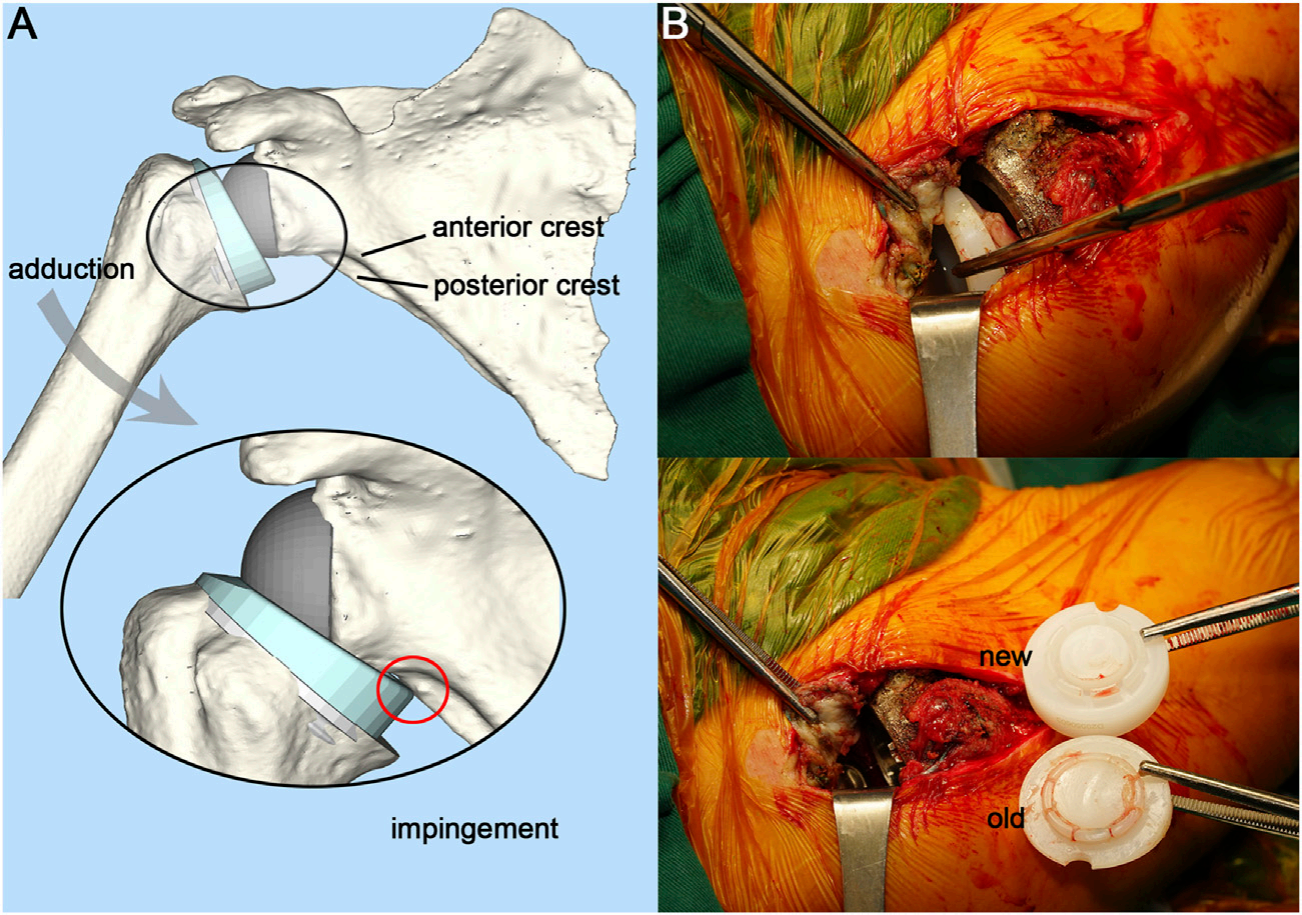
Illustration of adduction impingement and intraoperative photograph. **(A)**, Illustration of adduction impingement. **(B)**, Exposed joint cavity with visible polyethylene liner dissociation and intraoperative comparison of the new and the old liner.

Dislocation of the shoulder joint is a common complication after RTSA, sometimes accompanied by the dissociation between the polyethylene liner and the metal tray in some cases, which prevents closed reduction and necessitates operative revision. This poses significant challenges for surgeons (Nizlan et al., 2009; Patel et al., 2017; Paynter et al., 2020). There have been some case reports of polyethylene liner dissociation (Nizlan et al., 2009; Patel et al., 2017; Paynter et al., 2020). However, the biomechanical mechanism of liner dissociation is still unclear. In a case series of four patients with liner dissociation reported by Patel et al, (2017), the fixation structure of the liner was severely deformed, resulting in fixation failure. Patel speculated that the cause of the liner dissociation might be the impingement between the liner and the scapula (Figure 1A). Nevertheless, further mechanism investigation was not conducted. The liner dissociation also happened to one of our patients. The used polyethylene liner of the prosthesis had a similar claw-shaped fixation structure to that used in Patel’s case, which was found to be deformed (Figure 1B). There was a clinical study showed that 80% of late postoperative shoulder dislocations had evidence of adduction impingement and polyethylene liner failure (Kohan et al., 2017), so it is reasonable to suppose that the liner dissociation is related to the adduction impingement between the liner and the scapula.

To verify this hypothesis, observation of the liner fixation structure deformation during the adduction impingement is necessary. However, it is hard to capture the deformation *via* video radiography because of the precision limitation of the radiography system and the non-visualization of the polyethylene liner (Miranda et al., 2011; Patel et al., 2017). Computer simulation is a suitable method to investigate this question. Finite element analysis (FEA) has increasingly been used in the medical field, particularly in orthopedics where it can guide the design and optimization of prostheses (Liu et al., 2020; Zhang et al., 2021). Using this technique, we could dynamically simulate the impingement between the liner and the scapula, and precisely observe the deformation and the stress state of the polyethylene liner.

In this study, based on a case of shoulder dislocation with liner dissociation after RSTA, the FEA was selected to investigate whether adduction impingement is a cause of the liner dissociation of the prosthesis with this kind of claw-shaped fixation structure. The impingement between the polyethylene liner and the scapula during humeral adduction was dynamically simulated. Meanwhile, solutions to decrease the incidence of liner dissociation were also proposed.

## 2 Materials and methods

### 2.1 Clinical data

The patient was a 52-year-old male with a complaint of right shoulder joint pain and limited movement for two months. The patient had undergone an anatomical shoulder arthroplasty 11 years ago. Due to prosthesis loosening and limited shoulder movement, the patient underwent a customized 3D printed reverse shoulder prosthesis (AK Medical, Beijing, China) revision surgery 7 years ago. The neck shaft angle of the prosthesis was 140°. The baseplate was positioned neutrally and centrally on the glenoid. Detailed treatment information was presented in a case report (Zou et al., 2018). The postoperative functional examination of the shoulder joint revealed that when the patient’s arm was adducted close to the chest wall, the deltoid muscle was tense due to stretching. This is caused by the leverage generated by the contact between the liner and the scapula. At subsequent follow-up, the patient reported feeling friction within the joint during the walking arm swing. These demonstrated the presence of impingement and friction between the polyethylene liner and the scapula. The patient now has shoulder pain and limited movement. X-rays showed that the right shoulder was dislocated. After clinical evaluation, the surgeon decided to perform revision surgery.

### 2.2 Surgical procedure

The patient was placed in the left lateral position, and the right shoulder joint was routinely disinfected with iodophor. The right shoulder joint was incised from the rostral process, along the anterior border of the deltoid muscle, and ends at the humeral stop of the deltoid muscle, about 15 cm long. The patient had intact infraspinatus, teres minor, and teres major muscles. There was a moderate tear in the supraspinatus and subscapularis muscles. The deltoid muscle was separated under direct vision, and the anterior portion of the humeral shaft was incised at its stop to prevent damage to the axillary nerve branches. The short head of the biceps tendon was held medially, and the shoulder joint was flexed to reveal the joint cavity. The polyethylene liner was found to be completely detached and was removed. The fixation claws of the old liner had deformed and contracted towards the middle disc-shaped protrusion (Figure 1B), which caused the fixation claws to fail to snap into the metal tray. The scapular component of the prosthesis was properly polished and fitted with a suitable new liner. Accordingly, the humeral component was cleaned and trimmed to the proper size. The shoulder joint was reset and had good mobility and stability. The postoperative X-ray showed a satisfactory position of the shoulder joint. The patient had satisfactory shoulder function and was able to complete daily life activities at the 3-month follow-up.

### 2.3 Finite element analysis

**FIGURE 2:**
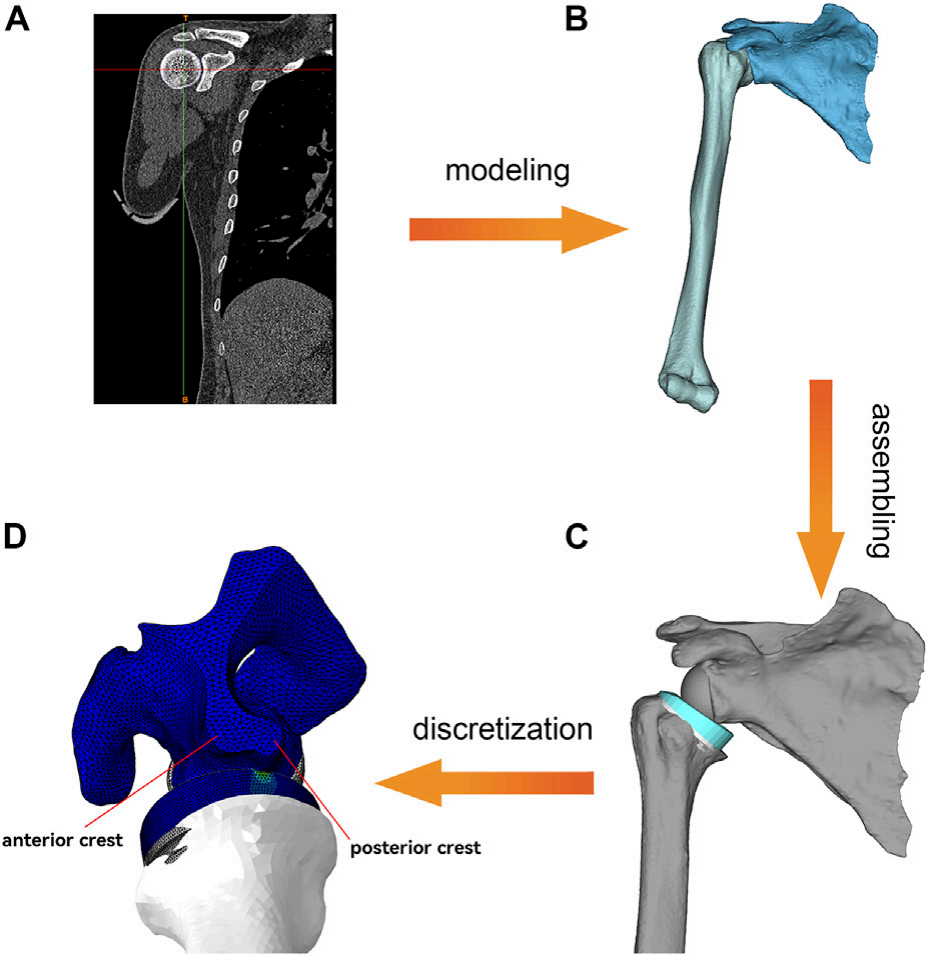
Overview of the FEA process. **(A)**, Shoulder CT data processing. **(B)**, Generation of scapula and humerus 3D models. **(C)**, Bone model and prosthesis model assembly and Meshing. **(D)**, Dynamic FEA. The lower border of the scapula is divided into a flat anterior crest and a narrow posterior crest.

A finite element (FE) model was developed to dynamically simulate the impingement between the polyethylene liner and the scapular pillar during humeral adduction. The entire experimental procedure is summarized in Figure 2. Supplementary Video S1 shows the impingement process and the Von Mises stress distribution.

#### 2.3.1 Model building

**FIGURE 3:**
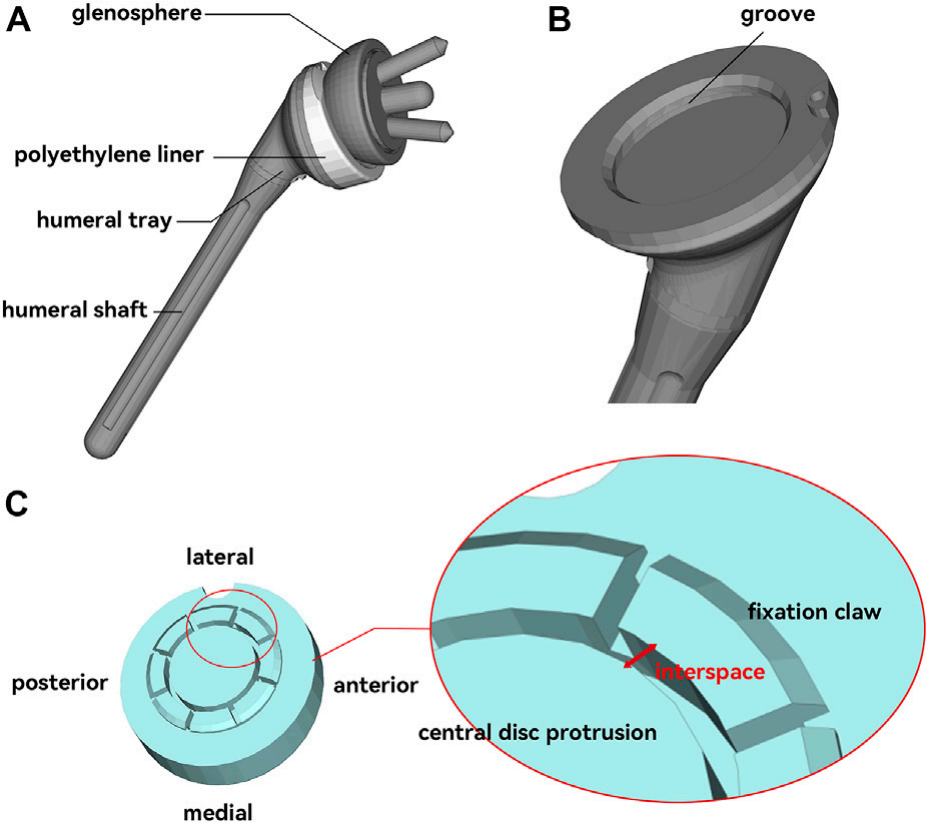
Reverse shoulder joint prosthesis model. **(A)**, Composition of the reverse shoulder joint prosthesis. **(B)**, Metal humeral tray with a groove that can catch the polyethylene liner. **(C)**, Polyethylene liner with eight symmetrical “claw” structures for fixation to the tray. The interspace between the claws and the intermediate disc-like protrusion was used as an indicator for this study.

This study was approved by the ethics committee of The Second Hospital of Jilin University approved. Informed consent was offered by the volunteer. The CT data of the volunteer’s shoulder were collected through the Philips iCT 256 CT scanner at 156 mA and 120 kVp with a slice thickness of 0.602 mm and exported in DICOM format. MIMICS 21.0 (Materialise, Belgium) was used to reconstruct the shoulder joint. Then the shoulder joint model was exported in standardized trigonometric language (STL) format. The medial part of the scapula was truncated to improve the simulation efficiency. The type of prosthesis was customized to the shape of the patient’s bone defect and 3D printed by the prosthesis manufacturer (AK Medical, Beijing, China). Its fixation structure was the claws on the underside of the liner (Figure 3C). The reverse shoulder prosthesis used in this study consisted of a scapular component and a humeral component. The scapular component included the glenoid base and glenosphere. The humeral component included the polyethylene liner, metal tray, and shaft (Figure 3). The assembly of the prosthesis and bone was performed under the supervision of an experienced surgeon. Both the humeral component and the glenosphere were placed in a neutral position without lateralization or tilt.

#### 2.3.2 Meshing and boundary condition setting

For discretization, all components were imported into Hypermesh 2020 (Altair Engineering, Troy, MI, United States) software. The components were divided into tetrahedral meshes of different sizes. A convergence test was performed to ensure the precision of the analysis. Three mesh sizes (1.5 mm, 1.2 mm, and 1.0 mm) of the liner were tested. The stress results difference for 1.2 mm and 1.0 mm was 1.1%. Four mesh sizes (4 m, 3 mm, 2.5 mm, and 2.0 mm) of the scapula were tested. The stress results difference for 2.5 mm and 2.0 mm was 6.0%. The mesh size of 1.0 mm for the liner and 2.0 mm for the scapula could meet the accuracy requirements of the analysis. The mesh size of the scapular component, glenosphere, and humeral shaft was set to 2 mm. Due to the small features and the region of interest to observe the stress distribution, the mesh size of the polyethylene liner and the humeral tray was refined to 0.75 and 1 mm, respectively.

The model was then imported into Abaqus 2021 (Dassault Systèmes, France) for the boundary conditions setting. Contact between the glenosphere and the shoulder glenoid, as well as the contact between the humeral shaft and the metal tray, were set to tie constraint (no relative movement was allowed between the parts in contact). All other surface contacts were set to be friction contacts with a friction coefficient of 0.07 (Quental et al., 2015). Because of the large difference in the elastic modulus between the metallic components and the polyethylene, all metallic materials were set up as analytical rigid bodies to improve the simulation efficiency. The material property of polyethylene liner was defined as isotropic linear elastic. The Young’s modulus was 850 MPa, the Poisson’s ratio was 0.44, and the density was 0.94 g/cm^3^. The Poisson’s ratio value used for bone was 0.3. The density (ρ) and the elastic modulus (E) of the bone were calculated from the following formulas based on the grayscale values (HU) of the CT images (Mo et al., 2019):

$$\rho \left(g/m^{3}\right)=-13.4+1017\;HU$$ (1)

$$E\left(Pa\right)=-388.8+5925\rho \left(g/m^{3}\right)$$ (2)

**FIGURE 4:**
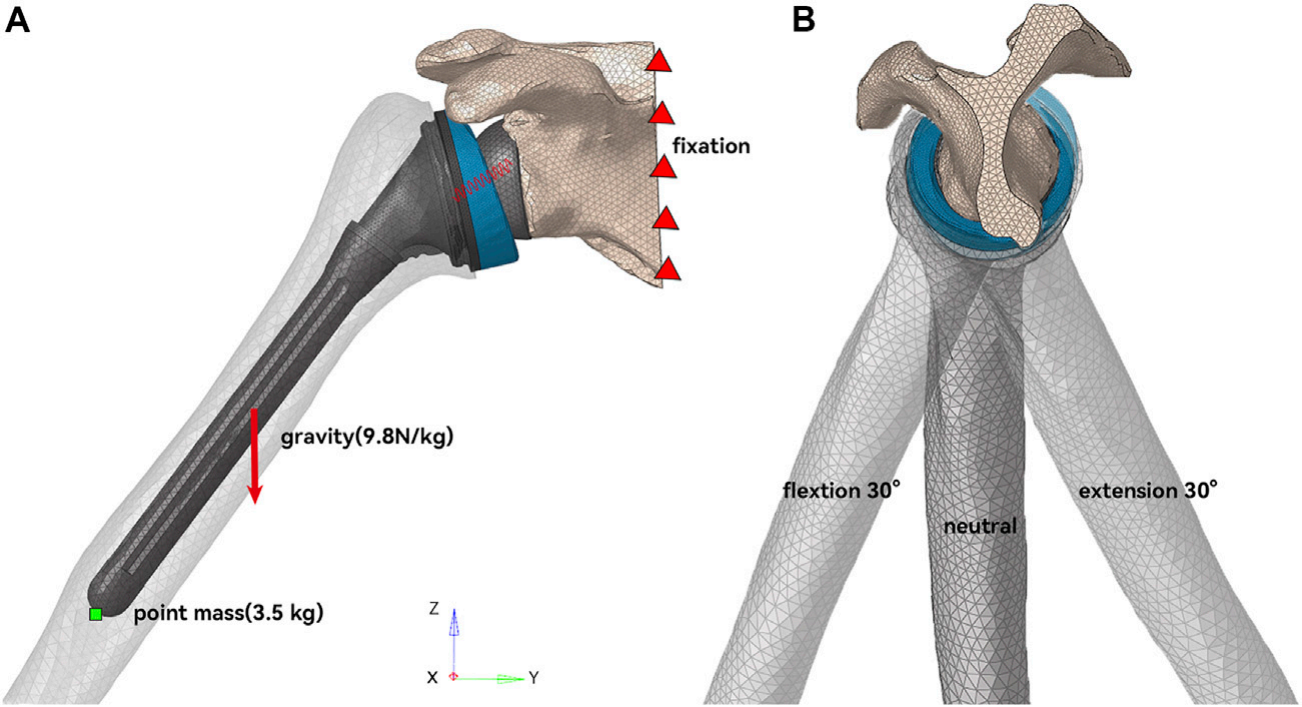
Schematic diagram of model working condition setting. **(A)** In the coronal plane, the humerus was initially abducted at 40°. **(B)** In the sagittal plane, the humerus had three initial postures of neutral, 30° flexion, and 30° extension.

The rotator cuff in the shoulder joint aids in internal rotation, external rotation, and abduction. It also squeezes the humeral head toward the scapular glenoid to stabilize the shoulder joint and prevent dislocation (Ackland et al., 2015). Since the active force processes of the muscles were not involved in this study, the rotator cuff was simplified to two nonlinear springs assemblies with anterior-posterior symmetry, which served to stabilize the shoulder joint and prevent dislocation (Hettrich et al., 2015) (Figure 4A). A 3.5 kg point mass was placed at the end of the humeral shaft (near the arm’s center of gravity) to represent the arm’s weight. The initial posture of the humeral component was set to 40° of abduction (Figure 4A). Except for the neutral initial posture, 40° abduction accompanied by 30° flexion or extension was also simulated (Figure 4B) to investigate the effect of different arm initial postures on the impingement. The medial plane of the scapula was set to be fully fixed, and then a gravitational acceleration of 9.8 m/s^2^ was applied to the entire system. The entire simulation was performed as an explicit dynamic analysis with an analysis time of 0.4 s. The arm was simulated to adduct under gravity and impinge. In addition, the cases of 10* and 0.1* elastic modulus of the liner were simulated to study the effect of liner material stiffness.

#### 2.3.3 Evaluation indicators

The Von Mises stress at the impingement location and the maximum principal stress (MPS) on the polyethylene liner were obtained. The Von Mises stress was used to assess the material failure of the polyethylene liner and the bone. The MPS was obtained to show the tendency of the fixation claw’s deformation. The interspaces between the fixation claws and the central disc protrusion before and after impingement were also recorded and analyzed to quantify the degree of deformation (DOD) of the fixation claw (Figure 3C). It was measured at four randomly selected points on each claw. The DOD was defined as the percentage change in the interspace before and after the impingement, which could be expressed as the following formula:

$$DOD=average\left(\frac{intb-inta}{inta}*100\%\right)$$ (3)

where *intb* was the interspace before impingement, *inta* was the interspace after impingement.

## 3 Result

### 3.1 Von Mises stress of the impingement point

**FIGURE 5:**
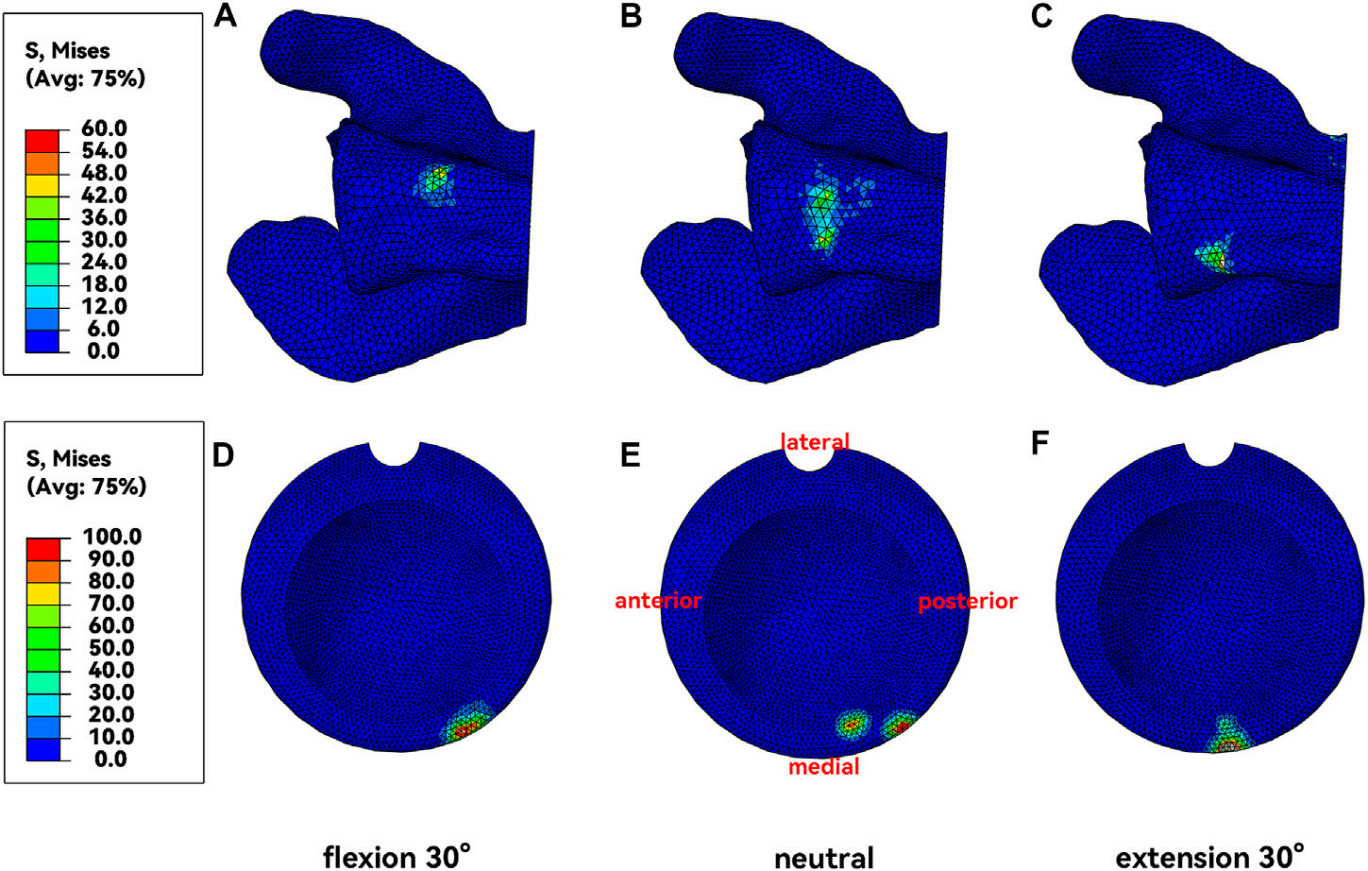
Von Mises Stress at the impingement point. **(A&D)**, Neural posture. **(B&E)**, Flexion posture. **(C&F)**, Extension posture.

The Von Mises stress at the impingement point in neutral, flexion, and extension humeral postures is shown in Figure 5. In the neutral posture, the liner impinged on both the anterior and posterior crest of the scapular pillar, with a maximum stress of 120 MPa on the scapula. There were two impingement points on the liner, located posteromedially, with a maximum stress of 145.7 MPa at the impingement point. In the 30° flexion posture, the impingement point for the scapular pillar and the liner was the anterior crest and posteromedial of the liner. The maximum stress on the scapula and the liner were 125.4 MPa and 152.9 MPa, respectively. In the 30° extension posture, the impingement point for the scapular pillar and the liner was the posterior crest and medial of the liner. The maximum stress on the scapular pillar and the liner were 160.7 MPa and 211.3 MPa, respectively.

### 3.2 Maximum principal stress distribution in fixation claws

**FIGURE 6:**
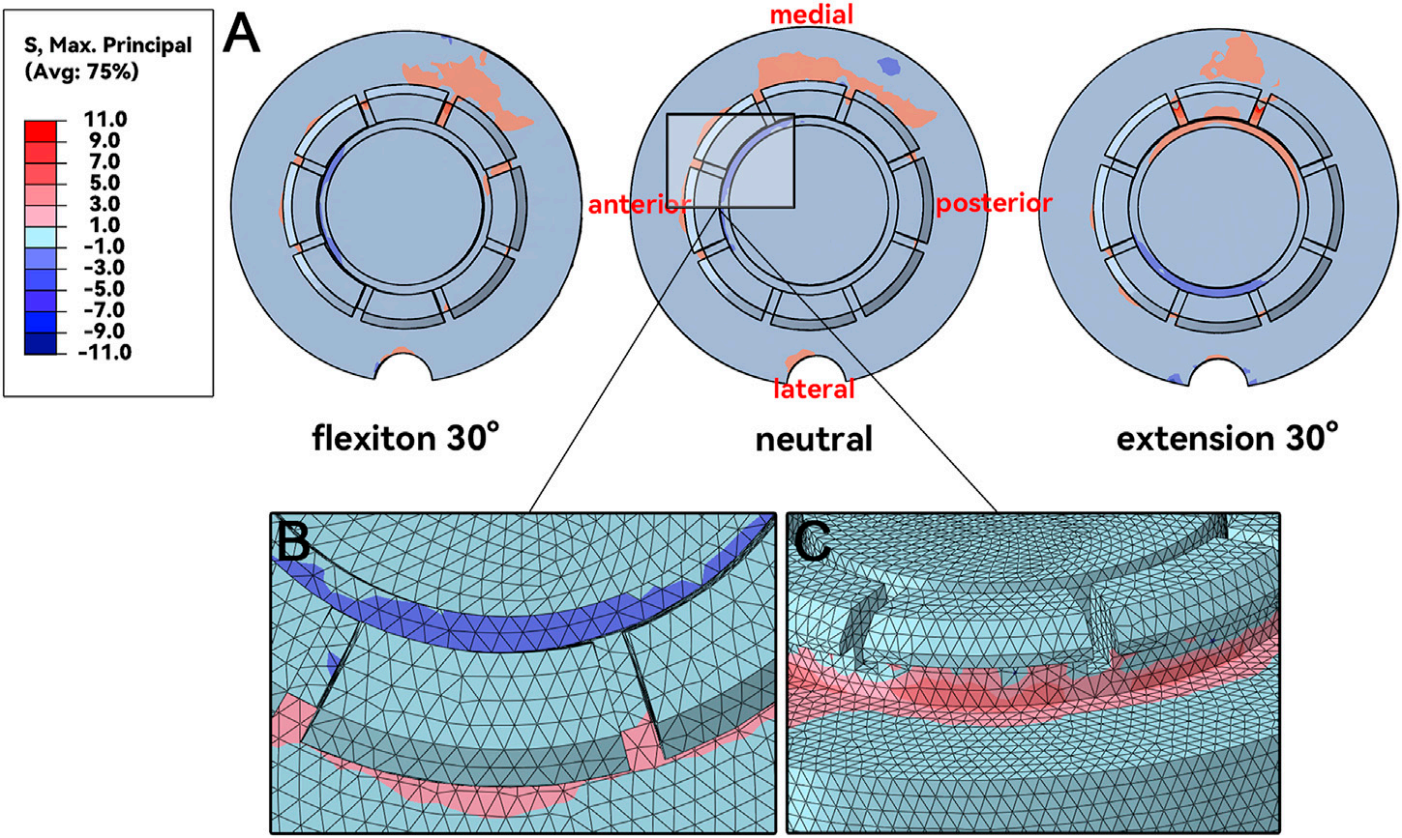
MPS at fixation claws. **(A)**, maximum principal stresses distribution. **(B&C)**, details of the inner side and outer side of the connection area.

Unlike the impingement point, the magnitude of the stress value on the fixation claws was much smaller, with the absolute value mostly less than 5 MPa (Figure 6). In the connection area between some fixation claws and the liner, compressive stress on the inside (Figure 6B) and tensile stress on the outside (Figure 6C) were observed. It represented a tendency for this part of the fixation claws to deform inward during liner impingement. Such regions were observed in the liner’s anterior-medial, anterior, and lateral positions when the humerus was in neutral, flexion, and extension postures, respectively (Figure 6A).

### 3.3 Degree of deformation

**FIGURE 7:**
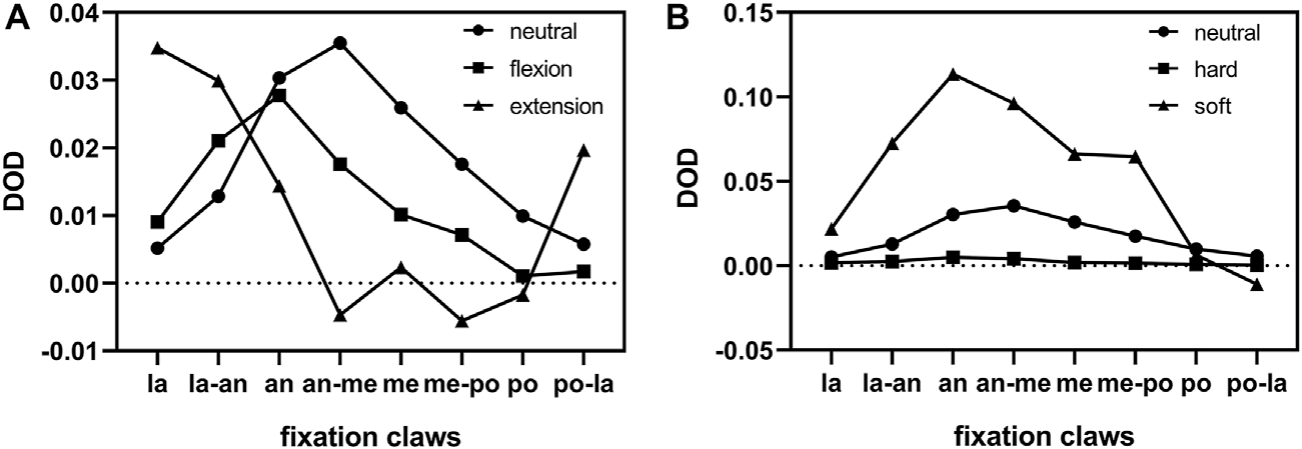
Distribution of DOD for different fixation claws. **(A)**, Comparison of different humeral postures. **(B)**, Comparison of different liner stiffnesses. la = lateral, an = anterior, me = medial, po = posterior.

The DOD results are shown in Table 1 and Figure 7, consistent with the fixation claws’ MPS results. The DOD was relatively large in the area where high MPS occurred. In neutral, flexion, and extension postures, the maximum DOD were 3.6%, 2.8%, and 3.5%, respectively (Figure 7A). Changing the elastic modulus of the liner could affect the deformation of the fixation claws (Figure 7B). In the neutral initial posture, when the elastic modulus of the liner was multiplied by 10, the maximum DOD was only 0.51%. Furthermore, due to the significant elastic modulus difference between cortical bone and UHMWPE, the maximum Von Mises stress at the scapular impingement point was 155.8 MPa, similar to the results of normal liner stiffness. When the elastic modulus was reduced by 10 times, DOD reached 11.4%. The maximum Von Mises stress at the scapular impingement point was 45.8 MPa.

**TABLE 1:** DOD*100% for different fixation claws.

	Neutral	Flexion	Extension	High elastic modulus	Low elastic modulus
**Lateral**	0.52	0.90	3.48	0.17	2.20
**Lateral-anterior**	1.28	2.10	2.99	0.26	7.25
**Anterior**	3.04	2.78	1.44	0.51	11.36
**Anterior-medial**	3.55	1.76	−0.47	0.42	9.63
**Medial**	2.59	1.02	0.24	0.19	6.62
**Medial-posterior**	1.76	0.71	−0.56	0.15	6.48
**Posterior**	0.99	0.11	−0.17	0.09	0.64
**Posterior-lateral**	0.58	0.17	1.97	0.04	−1.10

## 4 Discussion

Polyethylene liner wear and deformation is one of the most common causes of late failure after reverse shoulder arthroplasty, which originates from the impingement of the scapula and the polyethylene liner (Friedman et al., 2019). Dislocation of the shoulder joint after RTSA causes great trouble when accompanied by the dissociation of the polyethylene liner (Garberina and Williams, 2008; Nizlan et al., 2009; Patel et al., 2017). In this study, FEA was used to investigate whether the liner dissociation of the prosthesis with this kind of claw-shaped fixation structure was related to adduction impingement.

Most FEA studies of reverse shoulder prosthesis used the static analysis method, so the role of inertia was not essential and could be ignored (Denard et al., 2017; Ingrassia et al., 2018; Zhang et al., 2020). However, the impingement is a high-speed and dynamic process, and a fully dynamic finite element model is necessary (Cooper et al., 2019). It is tough to apply muscle forces with constantly changing magnitude and direction during this fully dynamic process. Therefore, the process of arm adduction under gravity and without the active muscle forces was simulated in this study. Nevertheless, in real life, the adduction of the arm is often accompanied by muscle force control, making the impingement of the scapula with the liner less intense than under the action of gravity alone. Thus, the stress and deformation due to the impingement obtained were higher due to the neglect of muscle forces.

The acquired results indicated two impingement points on the liner when the humerus was in neutral posture during the adduction process (Figure 5B). This was due to an anatomical detail that was easily overlooked. The lower edge of the scapular pillar is divided into two crests: the anterior and posterior crest, where the anterior crest is flatter, and the posterior crest is narrower (Figures 1A, Figure 2D). When the humerus adducted from the neutral posture, the liner impinged with both crests. The impingement points were located on the posteromedial side (Figure 5A), which was consistent with previous clinical studies (Lewicki et al., 2017). The impingement point of the liner was directly medial (Figure 5F) only when the humerus was in extension posture during adduction. At this time, the liner impinged with the narrower posterior crest, resulting in a stress of 160.7 MPa, higher than that of neutral posture and flexion posture. Nevertheless, humerus adduction from extension posture is less common in daily life than neutral and flexion posture.

Whether the impingement started from flexion, extension, or neutral posture inward, the stress generated on the liner exceeded 100 MPa, far beyond the yield stress of the UHMWPE (Pascual et al., 2012). There was no obvious difference in the magnitude of impingement stress generated by different impingement postures. Even though the real-life impingement with muscle control might not be that fierce, the liner was easily damaged. The maximum stress at the impingement point of the scapula reached 160 MPa in the extension posture and 120 MPa in both the flexion and neutral postures, which is less than the yield stress of the cortical bone (Heiner, 2008). According to these results, we might suspect that the scapular notch was not generated based on direct stress damage brought by adduction impingement, but based on the frictional wear. The particle debris produced by polyethylene wear was reported to contribute to the generation of the scapular notch (Levigne et al., 2008). The mechanism of the scapular notch generation needs further study.

The locations and the stress of the impingement points were discussed above. However, the direct reason for the liner dissociation was the failure of the fixation structure. In order to reflect the deformation direction of the fixation claws, the MPS was chosen to demonstrate the stress state of the liner fixation structure. When the stress on the structure was positive or negative, it meant that the structure was under tension or compression. The MPS distribution indicated that some fixation claws tended to deform inward. In the adjacent area between the fixation claws and the liner, the inner side was subjected to compressive stress while the outer side was subjected to tensile stress (Figures 6B,C). The fixation claws with high MPS varied when the impingement postures varied (Figure 6A). It is worth mentioning that the stress on the fixation claws did not reach the yield stress (Pascual et al., 2012), so the fixation claws did not enter the plastic deformation stage. The deformation might be the creep deformation caused by long-term stress (Glyn-Jones et al., 2008). A study of the creep behavior of polyethylene liner in hip prosthesis showed that creep deformation accounted for a large portion (up to 63%) of polyethylene liner deformation during gait simulation (Penmetsa et al., 2006). However, the creep behavior of polyethylene liner in reverse shoulder prosthesis has not been studied.

The MPS distribution was verified by the DOD results of the fixation claws. Figure 7 shows that most fixation claws deformed inward after the impingement. The areas with the greatest deformation were those with high MPS. The location where the maximum deformation occurred changed with the impingement posture, which explained the deformation of almost all fixation claws in our clinical case. Our arms are regularly in a neutral or flexion posture in daily life. Thus, when the impingement occurs in these postures, the fixation claws’ anterolateral, anterior, and anterolateral regions are more prone to deform (Figure 6A). In a case reported by Patel et al., a portion of the fixation claws were deformed (Patel et al., 2017). However, it is unknown whether this was the anterior fixation claw from the pictures in the paper. Therefore, the results above need to be validated by more multicenter long-term follow-ups.

To find a solution to reduce the incidence of liner dissociation, whether changing the elastic modulus of the liner material contributes to reducing the deformation of the liner fixation structure was investigated. As we know, the higher the elastic modulus of a material, the smaller its deformation at the same state of stress. However, the results of our analysis showed that increasing the elastic modulus of the polyethylene liner also increased the stress on its fixation claws. So, the effect of the change in the elastic modulus on the deformation of the fixation structure is worth exploring. The elastic modulus of the liner was increased or decreased tenfold, respectively. The results showed that changing the elastic modulus of the liner material could affect the deformation of the liner (Figure 7A). After the elastic modulus of the liner was multiplied by 10, the average deformation of the fixation claws was reduced to only 12.9% of the original. At the same time, the maximum impingement stress on the scapula was 155.8 MPa, which increased by 29.8% compared to the original. Based on this result, ideas for liner improvement could be proposed: we could improve the elastic modulus of UHMWPE by modifying the manufacturing process to decrease the deformation of the fixation structure (Ansari et al., 2016). We could use ceramic material, which also significantly reduces the osteolysis from debris generation (Sonntag et al., 2012). Besides, the claw-shaped fixation structure may be one of the important reasons for deformation and causing dissociation of the liner, and further research on improved fixation structure is needed.

In addition to improving the material of the liner to reduce liner dissociation, changing the shape parameter and placement of the prosthesis to reduce the impingement is also a viable option and has been studied extensively (Friedman et al., 2019). Reducing the neck shaft angle of the humeral component, and inferior overhang/inferior tilt/lateral offset of the glenosphere could reduce the impingement and improve the adduction angle (Gutierrez et al., 2008; Roche et al., 2013; Langohr et al., 2016). There are also eccentric glenospheres designed to increase inferior overhang while keeping screw fixation in the glenoid (Poon et al., 2014). However, some alterations might have a negative impact at the same time. Inferior tilt of the glenosphere would cause uneven distribution of the joint reaction load compared to the neutral tilt (Gutierrez et al., 2011). An excessive inferior overhang of the glenosphere might cause hypertonicity of the deltoid muscle (Friedman et al., 2019). Reducing the neck shaft angle of the humeral component causes the glenosphere to contact at the edge of the polyethylene liner, increasing contact pressure and increasing the generation of wear particles (Langohr et al., 2016). Therefore, the surgeon has to choose the appropriate prosthesis and its placement according to the anatomical characteristics of the patient’s shoulder.

Some aspects of this study need to be improved in the future. Firstly, the UHMWPE was defined as a linear elastic material. Since the stress at the impingement point already exceeded the yield stress of UHMWPE, the linear elastic material did not fully reflect the properties of UHWPMPE. However, the stresses of the fixation claws, which were the focus of this study, did not exceed the yield stress. Liner elastic material definition was enough and would not affect the significance of the results. Secondly, this study was based on a clinical case, and the results acquired in this simulation study need to be validated by more multicenter long-term follow-ups.

## 5 Conclusion

In this paper, based on a case of dissociation of the polyethylene liner in a reverse shoulder prosthesis, the adduction impingement of the liner and the scapular pillar was analyzed using finite element analysis, and the results showed contraction and deformation of the fixation claws of the liner, consistent with the case observation. Thus adduction impingement may be one of the reasons for the dissociation of the liner with this claw-shaped fixation structure. Creep might be one of the mechanisms for the permanent deformation of fixed structures, however, further experimental verification is needed. The stiffness of the liner material could affect the degree of deformation of the fixation claws. In the future, we can prevent liner dissociation by reducing the occurrence of impingement (by modifying the prosthesis design) or by strengthening the liner material.

## Data Availability

The raw data supporting the conclusion of this article will be made available by the authors, without undue reservation.
